# Buccal mucosa carcinoma: surgical margin less than 3 mm, not 5 mm, predicts locoregional recurrence

**DOI:** 10.1186/1748-717X-5-79

**Published:** 2010-09-15

**Authors:** Wen-Yen Chiou, Hon-Yi Lin, Feng-Chun Hsu, Moon-Sing Lee, Hsu-Chueh Ho, Yu-Chieh Su, Ching-Chih Lee, Chen-Hsi Hsieh, Yao-Ching Wang, Shih-Kai Hung

**Affiliations:** 1 Department of Radiation Oncology, Buddhist Dalin Tzu Chi General Hospital, Chiayi, Taiwan; 2 School of Medicine, Tzu Chi University, Hualien, Taiwan; 3 Department of Otolaryngology, Buddhist Dalin Tzu Chi General Hospital, Chiayi, Taiwan; 4 Department of Hematological Oncology, Buddhist Dalin Tzu Chi General Hospital, Chiayi, Taiwan; 5 Department of Radiation Oncology, Far Eastern Memorial Hospital, Taipei, Taiwan; 6 Department of Radiation Oncology, China Medical University Hospital, Taichung, Taiwan; 7 School of Medicine, Graduate Institute of clinical Medical Science, China Medical University, Taichung, Taiwan

## Abstract

**Background:**

Most treatment failure of buccal mucosal cancer post surgery is locoregional recurrence. We tried to figure out how close the surgical margin being unsafe and needed further adjuvant treatment.

**Methods:**

Between August 2000 and June 2008, a total of 110 patients with buccal mucosa carcinoma (25 with stage I, 31 with stage II, 11 with stage III, and 43 with Stage IV classified according to the American Joint Committee on Cancer 6^th ^edition) were treated with surgery alone (*n *= 32), surgery plus postoperative radiotherapy (*n *= 38) or surgery plus adjuvant concurrent chemoradiotherapy (*n *= 40).

Main outcome measures: The primary endpoint was locoregional disease control.

**Results:**

The median follow-up time at analysis was 25 months (range, 4-104 months). The 3-year locoregional control rates were significantly different when a 3-mm surgical margin (≤3 *versus *>3 mm, 71% *versus *95%, *p *= 0.04) but not a 5-mm margin (75% *versus *92%, *p *= 0.22) was used as the cut-off level. We also found a quantitative correlation between surgical margin and locoregional failure (hazard ratio, 2.16; 95% confidence interval, 1.14 - 4.11; *p *= 0.019). Multivariate analysis identified pN classification and surgical margin as independent factors affecting disease-free survival and locoregional control.

**Conclusions:**

Narrow surgical margin ≤3 mm, but not 5 mm, is associated with high risk for locoregional recurrence of buccal mucosa carcinoma. More aggressive treatment after surgery is suggested.

## Background

The incidence of buccal mucosa carcinoma has rapidly increased in Taiwan in recent decades; major risk factors for this disease are smoking, alcohol drinking, and betel nut chewing[1-3]. In patients with buccal mucosa carcinoma, locoregional recurrence (rate, 30-80%) is the main cause of treatment failure[4,5]. Several predictive factors for locoregional recurrence have been reported: bone erosion or invasion, positive surgical margin, perineural infiltration or invasion, vascular invasion, lymph node involvement, and extracapsular extension of tumor from the involved lymph node[6].

To reduce the risk of locoregional recurrence, radical surgery plus postoperative radiotherapy (RT) has been recommended for locoreginally advanced disease [7-9]. More recently, two large-scale randomized trials by the Radiation Therapy Oncology Group (RTOG) and the European Organization for Research Treatment of Cancer (EORTC) have demonstrated definitive benefits of post-operative concurrent chemoradiotherapy (CCRT) after radical surgery in patients with high-risk head-and-neck cancers [10,11]. National Comprehensive Cancer Network treatment guidelines recommend post-operative CCRT for patients with positive surgical margin or nodal extracapsular extension. However, in our limited treatment experience, patients with close surgical margins still have a high risk of locoregional recurrence. In the literature, close surgical margins less than 3 mm[12] or 5 mm[13-15] have been reported to associate with a high risk of cancer recurrence. However, there is still no universally agreed on definition of close surgical margin in buccal mucosa carcinoma.

Hence, we conducted this study to explore the effect of close surgical margin on outcome in patients with buccal mucosa carcinoma; and more importantly, to define close surgical margins in these patients. The primary endpoint was locoregional disease control and the secondary endpoints were disease-free survival, disease-specific survival, distant-metastatic survival, and overall survival. Other prognostic factors were also analyzed.

## Methods

### Ethical considerations

The procedures we followed were in accordance with the ethical standards of the committee on human experimentation of our institution and with the Helsinki Declaration of 1975, as revised in 1983. This study was approved by the Institutional Review Board at Buddhist Dalin Tzu Chi General Hospital before this study was performed.

### Patients and stage classification

The records of 134 patients with buccal mucosa carcinoma, treated from August 2000 to June 2008, were retrospectively reviewed. All patients received definitive treatments and had no distant metastasis. Twenty-four patients treated with CCRT alone (n = 7), RT alone (n = 5), neoadjuvant therapy plus surgery (n = 6), or who had a synchronous second primary (n = 6) were excluded. Thus, the remaining 110 patients who underwent radical surgery with or without adjuvant treatments were analyzed. Cancer staging was classified according to the American Joint Committee on Cancer, the 6^th ^edition[16].

### Treatment modality

Radical surgery consisted of wide excision with or without flap reconstruction for primary tumor and of unilateral or bilateral radical neck dissection for neck disease management. Pathology reports were reviewed for prognostic factor analysis. Adjuvant treatments were started 4-6 weeks after surgery, if indicated. Adjuvant CCRT was indicated for positive margin, extracapsular nodal spread, or combined any other 2 risk factors, including perineural invasion, vascular permeation, pT3, pT4 or N (+) nodal disease. Adjuvant RT was indicated for single risk factor except positive margin and extracapsular nodal spread.

For 78 irradiated patients, post-operative Intensity Modulated Radiotherapy (IMRT) was carried out using an inverse planning system (PLATO, Nucleotron Inc., Veenendaal, The Netherlands). The radiation field encompassed the surgical bed of the primary tumor and neck. The critical normal structures used for optimization included the brain stem, spinal cord, parotid glands, optic nerves, optic chiasm, lenses, and eyeballs. During RT, electronic portal imaging was performed weekly for verification. The prescribed doses delivered by external beam RT were as follows: 70-72 Gy to the gross tumor volume; 60-66 Gy to the high risk nodal region; and, 50-60 Gy to the low risk nodal region. Conventional RT fractionation was given, namely 1.8-2.0 Gy per day and 5 days per week for 6-7 weeks. The spinal cord dose was limited to 45 Gy.

Chemotherapy was given concurrently with and after RT, if indicated. The chemotherapy protocol consisted of a concurrent two-month course of cisplatin and fluorouracil (5-FU) followed by another 2-month course after RT, with regimens of cisplatin (60-100 mg/m^2^/day) on day 1 and 5-FU (1000 mg/m^2^/day) on days 1-5. We evaluated treatment toxicities by using the common toxicity criteria of the National Cancer Institute, V2.0[17].

### Statistical methods and definitions

Survival and follow-up times were calculated from the day of pathological diagnosis to the day of last follow-up or death. We used commercial statistical software (SPSS version 12.0; SPSS Inc., Chicago, IL, USA) to conduct statistical analyses, as follows: the Kaplan-Meier method to cumulatively estimate survival and disease-control rates; the log-rank test to assess curve difference between groups; Pearson's χ^2 ^test to evaluate differences between variables; and, Cox proportional hazard regression to perform multivariate analysis for hazard ratio (HR) assessment. For estimating the effective size, HR was provided with a 95% confidence interval (CI) in addition to a conventional *p *value. All tests were two-tailed and considered to be statistically significant when *p *< 0.05.

Surgical margin was defined as the distance between the outer edge of the tumor and the cut edge of the specimen under a microscope.

## Results

### Characteristics of patients

For all 110 patients, most patients were men (93.6%, 103/110), and 93 patients (84.5%) had a history of betel nut chewing. The treatment modalities were as follows: surgery alone (S), 29.1% (32/110); surgery plus post-operative RT (S + RT), 34.5% (38/110); and, surgery plus CCRT (S + CCRT), 36.4% (40/110). Table 1 shows the characteristics of the study participants and their tumors, and Table 2 shows the cancer stage distribution. After surgery, 56 patients had pathological stage I-II disease and 54 patients had stage III-IV disease. The incidences of neck nodal involvement were: 24% in all 110 patients, 20.0% in 70 pT1-2 patients, and 30% in 40 pT3-4 patients.

**Table 1:** Characteristics of 110 patients with buccal mucosa carcinoma.

Variable	Number of patients	%
Age		
≤50	44	40.0
>50	66	60.0
Gender		
Male	103	93.6
Female	7	6.4
pT		
pT1-2	70	63.6
pT3-4	40	36.4
pN		
pN0	84	76.4
pN1-3	26	23.6
Pathology stage		
I-II	56	50.9
III-IV	54	49.1
Histologic differentiation		
Well	6	5.5
Moderately	90	81.8
Poorly	12	10.9
NOS	2	1.8
Surgical margin		
Positive	6	5.5
Negative	104	94.5
Treatment		
S	32	29.1
S+RT	38	34.5
S+CCRT	40	36.4
Smoking		
No	16	14.5
Yes	93	84.5
Unknown	1	1.0
Betel nut chewing		
No	15	13.6
Yes	93	84.5
Unknown	2	1.8

Abbreviations: S, surgery alone; S+RT, surgery+radiotherapy; S + CCRT, surgery + concurrent chemoradiotherapy; NOS, not otherwise specified.

**Table 2:** Stage distribution in 110 patients with buccal mucosa carcinoma, n (%).

**Pathology stage (n, %)**		**pN0**	**pN1**	**pN2**	**pN3**
I (25, 22.7%)	pT1	25 (22.7)	1 (0.9)	1 (0.9)	0
II (31, 28.2%)	pT2	31 (28.2)	0	12 (10.9)	0
III (11, 10.0%)	pT3	9 (8.2)	1 (0.9)	4 (3.6)	0
IV (43, 39.1%)	pT4	19 (17.3)	2 (1.8)	5 (4.5)	0

#### Locoregional control and survival

**Figure 1:**
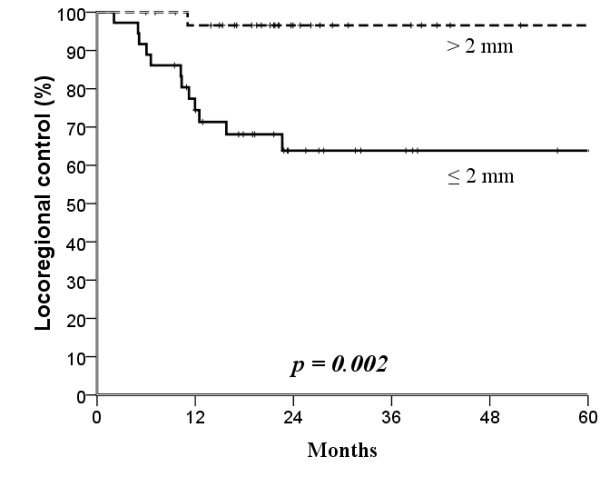
**Kaplan-Meier estimates of locoregional control over a 5-year period according to 2 mm cut-off surgical margins**.

**Figure 2:**
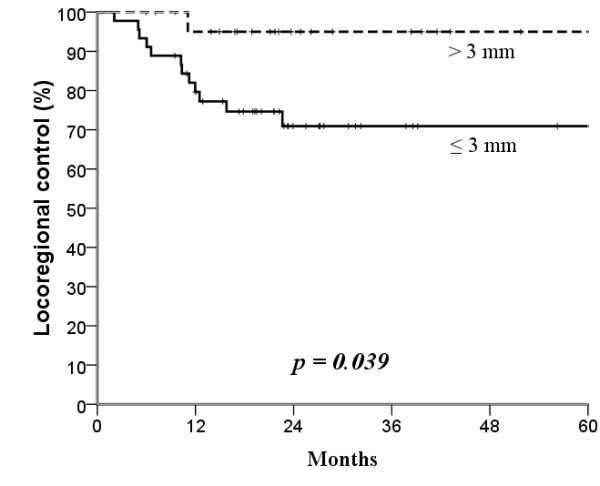
**Kaplan-Meier estimates of locoregional control over a 5-year period according to 3 mm cut-off surgical margins**.

**Figure 3:**
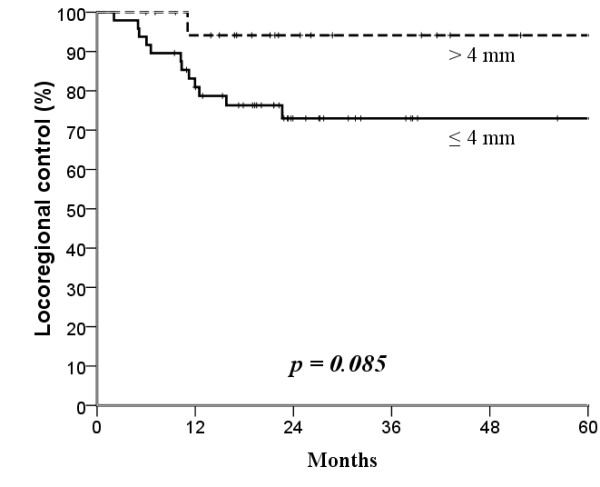
**Kaplan-Meier estimates of locoregional control over a 5-year period according to 4 mm cut-off surgical margins**.

**Figure 4:**
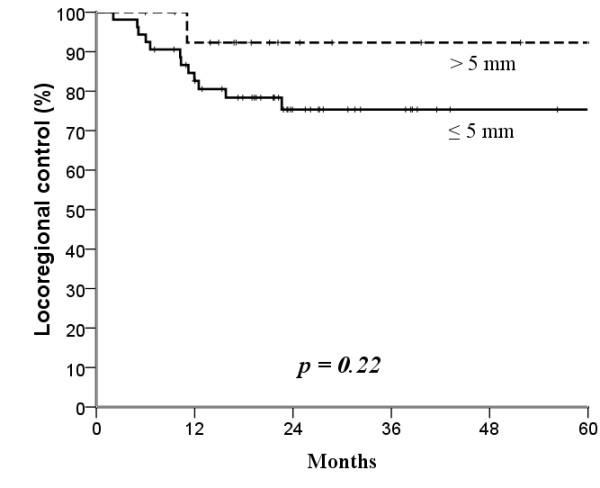
**Kaplan-Meier estimates of locoregional control over a 5-year period according to 5 mm cut-off surgical margins**.

The median follow-up time was 25 months (range, 4-104 months). The mean age was 53.7 years (range, 26-82 years). Surgical margin affected locoregional control: the narrower the surgical margin, the greater the difference in locoregional control after treatment (Figures 1, 2, 3 and 4; Table 3). Patients with surgical margin ≤3 mm had a statistically significantly higher risk for locoregional failure than those with surgical margin more than 3 mm; the 3-year locoregional control rates were 71% and 95%, respectively (*p *= 0.04). In multivariate analysis, surgical margin had a quantitative effect on locoregional control (hazard ratio [HR], 2.16; 95% confidence interval [CI], 1.14 - 4.11).

**Table 3:** The 3-year disease-free survival and locoregional control according to surgical margins

Surgical margin	Disease-free survival (%)	*p*	Locoregional control (%)	*p*
1 mm				
≤1 mm	56	0.02*	59	<0.01*
>1 mm	77		81	
HR, univariate	0.4 (95% CI, 0.19-0.86)	0.02	0.4 (95% CI, 0.16-0.80)	0.01
HR, multivariate	0.2 (95% CI, 0.06-0.72)	0.02	0.2 (95% CI, 0.05-0.67)	0.01
2 mm				
≤2 mm	59	<0.01*	64	<0.01*
>2 mm	93		97	
HR, univariate	0.1 (95% CI, 0.03-0.62)	<0.01	0.1 (95% CI, 0.01-0.64)	0.02
HR, multivariate	0.1 (95% CI, 0.01-0.60)	0.01	0.1 (95% CI, 0.01-0.72)	0.02
3 mm				
≤3 mm	67	0.06*	71	0.04*
>3 mm	91		95	
HR, univariate	0.3 (95% CI, 0.06-1.16)	0.08	0.2 (95% CI, 0.02-1.19)	0.07
HR, multivariate	0.2 (95% CI, 0.03-1.86)	0.17	0.3 (95% CI, 0.03-2.12)	0.21
4 mm				
≤4 mm	69	0.13*	73	0.09*
>4 mm	89		94	
HR, univariate	0.3 (95% CI, 0.08-1.49)	0.15	0.2 (95% CI, 0.03-1.53)	0.12
HR, multivariate	0.3 (95% CI, 0.04-2.16)	0.22	0.3 (95% CI, 0.04-2.52)	0.28
5 mm				
≤5 mm	72	0.36*	75	0.22*
>5 mm	86		92	
HR, univariate	0.5 (95% CI, 0.12-2.22)	0.37	0.3 (95% CI, 0.04-2.30)	0.25
HR, multivariate	0.5 (95% CI, 0.07-4.22)	0.56	0.6 (95% CI, 0.08-4.80)	0.63

Abbreviation: HR, hazard ratio; 95% CI, 95% confidence interval.

*, *p *values were calculated by using Kaplan-Meier method; other non-specified *p *values were calculated by using Cox proportional hazard regression.

For all patients, the rates of 3-year locoregional control, disease-free survival, disease-specific survival, distant metastasis-free survival, and overall survival were 73%, 70%, 84%, 96%, and 82%, respectively.

### Prognostic factors

For univariate analysis, pathology stage, pN classification, surgical margin, and nodal extracapsular spreading (ECS) were significantly associated with survival (Table 4). The pN classification and surgical margin also significantly affected locoregional control. The pN classification (pN0 *versus *pN1-3) and surgical margin (≤2 *versus *>2 mm) were the two most significant factors affecting clinical outcome. However, for surgical margin (cut off at 3 mm), the statistical significance of its association with locoregional control was only found at the clinical end point of 3 years.

**Table 4:** The 3-year clinical outcomes according to prognostic factors.

Factor	Overall survival (%)	*p*	Disease-specific survival (%)	*p*	Disease-free survival (%)	*p*	Locoregional control (%)	*p*	Distant metastasis-free survival (%)	*p*
Age										
≤50/>50	78/85	0.61	78/89	0.24	66/72	0.59	72/74	0.97	93/98	0.18
Gender										
Male/Female	84/51	0.1	86/60	0.32	71/42	0.51	74/56	0.9	97/80	0.14
pT										
pT1-2/pT3-4	87/75	0.08	89/79	0.15	72/66	0.65	73/73	0.88	98/92	0.13
pN										
pN0/pN1-3	96/33*	a	98/34*	a	81/32*	a	81/46*	a	100/79*	A
Pathology stage										
I-II/III-IV	98/66*	a	100/69*	a	81/58*	0.01	81/65	0.07	100/92*	0.04
Grade										
1/2+3	83/81	0.99	100/83	0.35	100/67	0.17	100/71	0.2	100/96	0.63
Surgical margin										
(+)/(-)	63/83	0.26	63/86	0.16	44/71	0.21	44/75	0.12	100/96	0.63
≤2/>2 (mm)	64/94*	0.02	66/97*	0.01	59/93*	b	64/97*	b	91/97	0.37
≤3/>3 (mm)	70/91	0.18	71/95	0.09	67/91	0.06	71/95*	0.04	93/95	0.74
ECS										
(+)/(-)	25/77*	a	33/77*	b	50/71*	0.01	75/73	0.62	75/76	0.02
PNI										
(+)/(-)	72/85	0.23	72/80	0.15	64/71	0.32	73/73	0.64	91/96	0.41
Bone invasion										
(+)/(-)	85/91	0.2	85/91	0.2	55/70	0.46	55/74	0.3	100/98	0.63
Skin invasion										
(+)/(-)	76/94	0.19	82/94	0.48	56/64	0.78	67/64	0.27	84/100	0.05

*, Statistically significant difference; a, *p *< 0.001; b, *p *< 0.01; PNI, perineural invasion; ECS, extracapsular spread; (+), positive; (-), negative.

In multivariate analysis, both pN classification and surgical margin independently affected disease-free survival and locoregional control. Furthermore pN classification also affected overall and disease-specific survivals (Table 5).

**Table 5:** Prognostic factors affecting clinical outcome in multivariate analysis.

Factor	HR (95% CI)	*p*
Overall survival		
Nodal status (pN0 *vs*. pN1-3)	27.1 (3.19-229.32)	<0.01
Disease-specific survival		
Nodal status (pN0 *vs*. pN1-3)	28.3 (3.33-241.53)	<0.01
Disease-free survival		
Nodal status (pN0 *vs*. pN1-3)	7.3 (2.11-25.44)	<0.01
Surgical margin (≤2 mm *vs*. >2 mm)	0.1 (0.01-0.60)	0.02
Locoregional control		
Nodal status (pN0 *vs*. pN1-3)	5.9 (1.59-21.92)	<0.01
Surgical margin (≤2 mm *vs*. >2 mm)	0.1 (0.01-0.72)	0.02

Abbreviations: HR, hazard ratio; 95% CI, 95% confidence interval.

## Discussion

### Synopsis of key findings

In this study, two major findings indicated that surgical margin ≤3 mm, not 5 mm, was a useful pathological parameter for predicting locoregional recurrence in patients with buccal mucosa carcinoma treated surgically. First, the 3-year locoregional control rates were significantly different at the cut-off value of 3 mm (≤3 *versus *>3 mm, 71% *versus *95%, *p *= 0.04), but not at 5 mm (75% *versus *92%, *p *= 0.22). Second, we found a quantitative correlation between surgical margin and locoregional failure (HR, 2.16; 95% C.I., 1.14 - 4.11; *p *= 0.019), which suggested that every 1 mm decrease in surgical margin significantly increased the rate of locoregional failure by 116%.

### Clinical applicability and comparison with other studies

For patients with buccal mucosa carcinoma and positive surgical margins, postoperative clinical outcome is poor [18,19]. For patients with close surgical margins, the risk for cancer recurrence is high [12,13,15]. However, how many millimeters between the tumor and edge of the specimen define a close surgical margin? More importantly, can this definition be used to make a treatment recommendation after surgery? The answers to these questions are still controversial. A previous study suggested 3 mm was adequate to reduce the risk of cancer recurrence [12], but most studies recommended 5 mm [13-15]. In our study, surgical margin ≤3 mm tightly associated with high locoregional recurrence rate in patients with buccal mucosa carcinoma. Considering survival as the endpoint, overall survival was significantly poorer in patients with surgical margin ≤2 mm than in those with margin >2 mm (Table 4). In our study, we also adjusted treatment modality. The treatment results did not have significant difference. For a close margin of ≤3 mm, more effective and safe drugs, re-surgery or higher doses of radiotherapy should be considered into multi-modal treatment strategy.

Thus, we would suggest that *for locoregional control*, surgical margin of 3 mm, not 5 mm, may be a suitable cut-off point to use for post-operative adjuvant therapy decision making; however, *for survival*, surgical margin of 2 mm may be the cut-off point at which stronger post-operative treatment is recommended.

Several other post-operative prognostic factors were evaluated in our study. In agreement with other studies [9,19-21], our study found that pN classification was the most important prognostic factor for both survival and locoregional control. The 3-year overall survival and locoregional control rates in patients with pN0 and pN1-3 diseases were 96%/33% and 81%/46%, respectively (both *p *values < 0.001; Tables 4 and 5), suggesting that intense post-operative adjuvant therapy should be given to patients with pN1-3 disease, and CCRT with or without targeted therapy in a clinical trial setting should be considered.

ECS of involved lymph nodes has been found to be a poor prognostic factor. Patients with both ECS and a positive surgical margin had significantly poorer overall survival than those without these risk factors [10,11,15]. In our study, ECS significantly associated with poor survival only in univariate but not in multivariate analysis.

### Strengths of this study

The main strength of this study is that the medical and surgical records were complete and the pathologies were well defined for all 110 patients included with buccal mucosa carcinoma treated with radical surgery; the homogeneity of this study population increases the clinical applicability of our results to such patients.

### Limitations of this study

This study had two main limitations: a retrospective design and small number of cases. Thus, the conclusions of this study should be confirmed by further investigations. Despite these limitations, our data showed that a surgical margin of more than 3 mm may be relatively safe margin in patients surgically-treated for buccal mucosa carcinoma.

## Conclusion

More aggressive post-operative therapy is suggested for patients with buccal mucosa carcinoma excised with a close margin of ≤3 mm.

## Competing interests

The authors declare that they have no competing interests.

## Authors' contributions

CWY, WYC and HSK developed the ideas for these experiments, performed much of the work, and drafted the manuscript. LHY, HFC, LMS, HHC, HCH and SYC designed the study, collected the data and interpreted the data. LCC performed the statistical analysis. All authors read and approved the final manuscript.
